# Going viral in rheumatology: using social media to show that mechanistic research is relevant to patients with lupus and antiphospholipid syndrome

**DOI:** 10.1093/rap/rky003

**Published:** 2018-03-05

**Authors:** Thomas C R McDonnell, Chris Wincup, Anisur Rahman, Ian Giles

**Affiliations:** Division of Medicine, Rheumatology, University College London, The Rayne Institute, 5 University Street, London WC1E 6JF, UK

**Keywords:** systematic lupus erythematosus and autoimmunity, antiphospholipid syndrome, patient attitude to health, study design, Social media

## Abstract

**Objectives:**

There is a lack of published data regarding patient interaction in basic scientific research, including methodologies for simple, cost-effective interactions and the outcomes of such studies. Therefore, we aimed to evaluate the ease of generating patient opinion data on specific scientific research projects whilst establishing a template for other groups to follow. Our secondary objective was to assess which research topics are of most interest to patients with SLE and/or APS.

**Methods:**

Through patient-based interactions, we developed a lay summary of a mechanistic research proposal and a set of associated questions to assess patient opinion on this research topic. We disseminated the questions as an online survey with associated lay summary through patient-based charity websites and social media. The survey was open for 3 weeks.

**Results:**

Of 527 respondents, 520 reported having SLE or APS. The patient response to the research proposal was overwhelmingly positive, with the majority expressing strong interest in the mechanistic aspect of the project. Analysis of free text box responses confirmed that the most popular research topics for patients were as follows: treatment, genetics, triggers, diagnosis and mechanistic research. Interestingly, patient interest in disease mechanisms featured more frequently than clinical topics, such as management of disease flares.

**Conclusion:**

It is possible to conduct short-term, valuable patient engagement at low cost, using an online survey and social media. This methodology may form a good template for future patient engagement. The volume and distribution of positive response shows that patients are interested in mechanistic research.

Key messages

- Social media is a powerful, cheap and effective tool for patient and public engagement.
- Patients with SLE/APS are interested in mechanistic approaches to research.
- Large amounts of relevant feedback can be collected rapidly from patients with rheumatic diseases.

## Introduction

Patient and public involvement (PPI) is considered a cornerstone of clinical research [1], enabling researchers to identify and address questions most relevant to patients [2, 3]. In contrast, PPI in mechanistic research is less common, primarily owing to perceived challenges among researchers [4]. In particular, PPI is often considered expensive and time consuming, and in diseases with a relatively low incidence, such as SLE, reaching a large audience can be challenging. In addition to these methodological barriers, there is a need for researchers to refine the science of patient input to produce quantitative data [5]. To overcome these problems, a template for quick, effective collection of measurable patient opinions is required. We set out to prove that the process can be inexpensive, expansive and inclusive.

Social media has become an established means of online communication over recent years. Platforms such as Twitter and Facebook allow users to publish their own content directly to a worldwide forum. The use of social media in the medical profession is also increasing. In 2014, a study reported that 72% of Canadian oncology physicians used social media regularly [6]. Furthermore, patients now frequently use social media to gain information and interact with online health communities and support groups, such as the APS Support UK community and various charity Twitter groups [7,8].

Therefore, we developed and undertook a PPI project with measurable outcomes using social media to gauge the relevance of our proposed mechanistic, non-clinical, basic science research into the effect of autoantibodies upon the interaction of complement and coagulant serine proteases in APS and SLE and, in doing so, to assess where patient interest lies in basic science research.

## Methods

### Study design

We designed a lay summary describing our mechanistic research proposal relevant to patients with SLE and/or APS and a nine-item questionnaire in the form of a survey to gauge the interest of patients in a range of related and more general research topics. The aim was to reach the maximal number of patients in the shortest possible time for minimal cost. We used social media and liaised with relevant patient-based charities (LUPUS UK and APS Support UK) to increase dissemination. Ethical review was not required because no patient-identifiable data were collected. We also accessed the UK Health Research Authority decision tool (available online at https://www.hra.nhs.uk/approvals-amendments/what-approvals-do-i-need/), which confirmed that ethical approval was not necessary for this study.

### Question design and lay summary

The proposed questionnaire and lay summary were refined through consultation with an expert patient, patient charity representatives. The survey was limited to nine questions to reduce the likelihood of participants abandoning it before completion. The first question aimed to identify patient disease groups, followed by questions about the importance of activity markers, of antibody testing for the patient and for the clinician and future therapeutic advances. To ensure quantitative data collection, questions were formatted to capture answers as either a rating of the importance of research questions on a numerical scale of 1–10 (with 10 being of highest importance) or to answer yes or no (Table 1). Qualitative data were also collected through a free text box, allowing participants to provide further detail of their opinions regarding research questions not answered elsewhere.

**Table 1:** The full questions and options of the questionnaire hosted online for patients

Question	Answers
How long have you had lupus and/or APS?	<5 years, 5–10 years, >10 years
How valuable do you think research to identify new blood tests to measure disease activity for lupus and APS is?	1–10
How valuable do you think it is to know if you are positive for autoantibodies that may influence your treatment?	1–10
How important do you think it is to know the effects your autoantibodies may have on your treatment?	1–10
Do you think it is important for doctors to know whether autoantibodies should influence treatment choices?	Yes/no
Would you take an anti-FXa drug if you thought it would help treat your lupus or APS?	Yes/no/if recommended by a clinician
Do you feel research like this is answering questions specific to you?	Yes/no
What research questions do you think we should investigate?	Free text

FXa: factor Xa.

### Response capture

The survey was hosted online (via the Survey Monkey website, www.surveymonkey.com) and circulated through commonly used social media platforms, namely Twitter (www.twitter.com) and Facebook (www.facebook.com). In addition, the lay summary was hosted with links to the survey on the charity websites of LUPUS UK (www.lupusuk.org.uk) and APS Support UK (www.aps-support.org.uk). The survey remained open for a 4-week period from 5 March 2017 to the 5 April 2017 (inclusive), during which patient responses were captured. To ensure the validity of data, individual Internet Protocol addresses were allowed a single submission, thus preventing multiple attempts by the same participant. A preliminary question asking the respondent to confirm a diagnosis of SLE and/or APS was included to reduce the risk of capturing data from other patient groups, healthy individuals or relatives.

### Analysis of response

Quantitative data were analysed using Microsoft Excel 2016 software. Qualitative data were reviewed and assigned to predetermined research categories by T.C.R.M. and checked for scientific/clinical accuracy by C.W. and I.G. Any disagreements were resolved by discussion, and where comments were felt to relate to more than one research category, the comment was placed in the appropriate number of relevant categories. Denominators relate to the total number of responses per question.

## Results

Total expenditure was £26, which covered the costs of accessing and downloading data collected from the online survey.

### Data capture

A total of 527 responses were captured across a 4-week period. Peaks of 104 and 152 responses per day on days 1 and 9 of the survey were correlated with the days on which the questionnaire was posted on different charity Twitter feeds. The original tweet on Twitter gathered 127 total engagements and was seen 2072 times on that platform.

### Disease duration

**Fig. 1:**
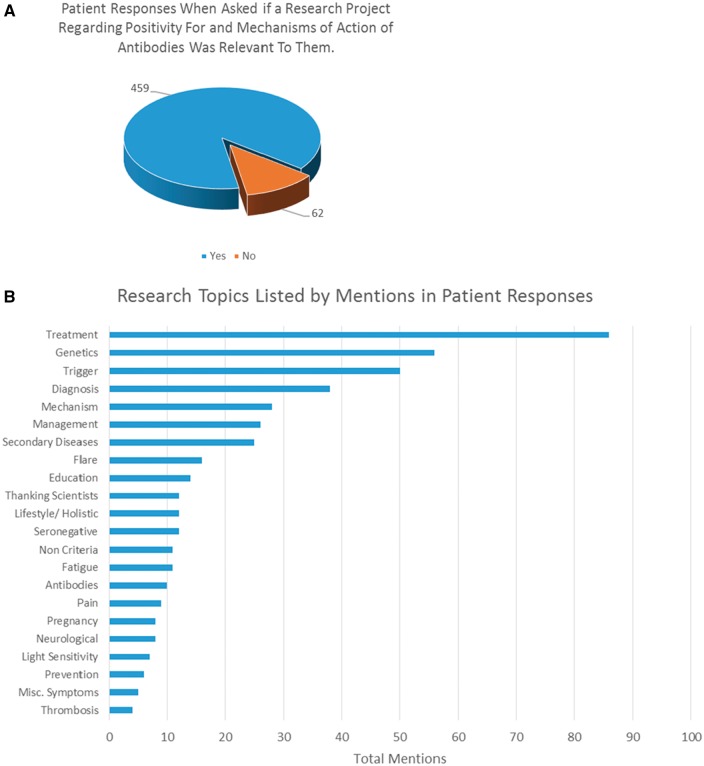
Research outcomes of patient engagement Various disease- and research-related outcomes are shown. (**A**) Responses from patients when presented with a research project regarding antibody positivity and potential mechanisms. (**B**) Research topics raised by patients and their frequency in the survey (the length of the bar).

Of the 527 responses, almost all (520, 98.7%) respondents confirmed having SLE and/or APS. The majority of patients had a disease duration of >10 years (255/520; 48%). Disease duration of 5–10 years was reported in 116/520 (22%), and those with a diagnosis of <5 years accounted for 149/520 (28%) of responses (Fig. 1A). The remaining 1.3% (7/527) of responders reported having neither condition, so their responses were excluded.

### Opinions on mechanistic research and autoantibodies

The lay summary described a project based on the study of antibodies to serine proteases, their potential role in SLE and APS pathogenesis and possible use as biomarkers of disease activity. When asked how valuable they considered this research into new blood tests for disease activity in lupus or APS, the average response was 9.5/10, with 87% (453/519) of responders scoring 9 or higher. Only four respondents gave a value below 5/10. Similar responses were seen for questions regarding how important they considered knowledge of their antibody status (9.5/10, 454/519, 87%) or the importance of clinicians knowing the effects that antibodies may have on informing treatment decisions (9.5/10, 456/518, 88%). Only two patients scored all three questions as <7/10.

### Considerations of new therapies

Regarding new treatments, we posed a question asking whether patients would take a serine protease blocking drug for treatment of SLE or APS. Only 1.4% replied negatively (7/517), with the remaining 98.6% (510/517) split between yes (41%, 212/517) and if recommended by a clinician (57.8%, 298/517). Eighty-eight per cent (454/514) of patients responded positively when asked whether research regarding antibody positivity and mechanisms of action was felt to be relevant to their disease (Fig. 1B).

### Other opinions

From 277 individual free-text responses, 464 comments were extracted. Ten were excluded for being questions about personal circumstances. The remaining comments were grouped into 22 categories according to content. These included treatment (18.9%, 86/464), genetics (12.3%, 56/464), triggers (11.0%, 50/464), diagnosis (8.3%, 38/464), mechanisms (6.1%, 28/464) and management (5.7%, 26/464). The full list of categories is summarized in Fig. 1B.

## Discussion

Overall, we found this PPI activity to be a simple, inexpensive and time-efficient process, confirming that patients are interested in mechanistic research. To date there is a surprising lack of published data regarding PPI in basic science projects regarding mechanistic studies. Often it is assumed that patients will find this sort of study less interesting or relevant when compared with clinical research; consequently, PPI may be overlooked.

The potential time and financial constraints involved in generating PPI are also commonly reported barriers. Various studies have attempted to address this imbalance [4] through several approaches, with varying degrees of success [9]; for example, Elwyn *et al*. [10] attempted to assess patient interest in research questions in asthma. They conducted a survey by mailing it to 1146 participants and posting it online on a relevant charity website for 3 weeks. The entire postal study was open for a total of 3 months. Of 370 responses, 211 were from the online website link and 103 responses were discarded because they were deemed to contain irrelevant information [10]. This study cost a total of £29 000 to conduct and demonstrates the potentially complex and costly nature of patient engagement. In contrast, we obtained 527 responses, discarded only seven and spent a total of £26 over a 3-week data collection period.

Through the use of social media, we have demonstrated that it is possible to canvas the opinions of a large group of patients in a short period of time at very little expense. The high volume of responses is even more remarkable considering the low prevalence of SLE. Conducting this survey on a face-to-face basis would have required considerably more time and cost.

In addition, our results have identified areas of interest to patients in SLE/APS that we assumed to be less relevant or important to patients because they do not directly translate into a new therapy. It was clear from free-text comments that patients are interested in mechanistic approaches to research as well as diagnostic and therapeutic approaches. This finding has not previously been reported. Patients also expressed an interest in genetic research, specifically regarding transmission of their diseases to their progeny. It should be noted here, however, that it is possible patient opinion has been influenced by the associated explanation and, as such, more in-depth research is required to confirm these trends.

To overcome problems that can occur owing to analysis of qualitative data, that is, misinterpretation and misunderstandings seen in the study by Elwyn *et al.* [10], patient contact and lack of patient understanding of the underlying research question, we provided a lay summary of the research that had been developed in collaboration with patient experts and relevant charities. In addition, we ensured that the results gained were predominantly binary or numerical, allowing for data analysis in a number of ways and ensuring a measurable outcome.

In contrast, data collected in the free text box were qualitative. To safeguard these data from misinterpretation, responses were analysed by one member of the team in order to assign the comments to categories before two clinicians independently checked and confirmed the correct scientific/clinical categorization. As with the study of Elwyn *et al.* [10], we found that some patients included comments relating to their personal circumstances, although at a much lower proportion in our study. This finding may be attributable to the more defined research project detail we supplied in the lay summary along with the questionnaire.

The responses we received were overwhelmingly positive, with 12 patients using the text box to comment on how beneficial it was to see such research being undertaken. Patients seemed enthusiastic to be involved, even re-posting the survey to message boards for other patients to access. The survey was also shared worldwide by patient associations, resulting in a global response.

This approach does, however, have limitations; for example, it relies on familiarity with information technology, the Internet and social media platforms. Furthermore, basic English language and literacy levels are required (although there is potential for future surveys to be translated). There is also the risk of targeting only the specific demographic of the population who regularly use social media. This risk might bias towards a younger population, who tend to use these platforms more than older patients. For instance, the office of government statistics states that in 2017 96% of people aged 16–24 years used social media platforms compared with only 68% aged 45–54 years (www.ons.gov.uk). In an attempt to account for this potential bias, we included a question regarding disease duration. Interestingly, the majority of responses were from patients with a disease duration of >10 years. Given that the mean age at which patients develop SLE or APS is ∼30 years, the majority of our respondents with disease duration of >10 years are likely to be in their fifth decade. Therefore, we do not think that we have encountered a younger age bias; however, without collecting age-of-onset data this is hard to prove conclusively.

Tunnicliffe *etal*. [11] recently published a study looking at the research priorities of young patients with SLE. Using face-to-face semi-structured interviews with 26 participants they identified seven themes and prioritization of research on alleviating poor psychological outcomes. Our research methods were very different from theirs in terms of the number of participants, broader age range and contact online rather than face to face. It is important to recognize that a mixture of different research methods, as exemplified by these two contrasting studies, should be used to investigate the important area of patient preferences and opinions regarding research in SLE.

We guarded against confounding by non-SLE/APS patients answering the survey by asking participants to self-confirm their diagnosis, and seven respondents answered that they had neither SLE nor APS. We designed the survey to make this question mandatory before completing the remaining questions. The one bias we could not guard against entirely was response bias [7]; however, research suggests that criticism may be raised against most questionnaire-based studies. Equally, it could be suggested that the context of the survey might have influenced question answers given the associated text; however, this would be true of any PPI undertaken in the context of a disease.

In conclusion, this PPI exercise demonstrates that a mechanistic research project is valuable and highly relevant to patients with SLE/APS. Furthermore, we demonstrate that a social media-based survey approach is a powerful tool, which enables the opinion of a large number of patients to be captured in a short time frame with minimal cost. This approach could be adopted by other groups, ensuring that patients are given an active role in directing future research.
